# Metabolic and Hepatic Effects of Energy-Reduced Anti-Inflammatory Diet in Younger Adults with Obesity

**DOI:** 10.1155/2021/6649142

**Published:** 2021-02-05

**Authors:** Gordana Kenđel Jovanović, Ines Mrakovcic-Sutic, Sandra Pavičić Žeželj, Indira Benjak Horvat, Lucia Šuša, Dario Rahelić, Sanja Klobučar Majanović

**Affiliations:** ^1^Department of Health Ecology, Teaching Institute of Public Health of Primorsko-Goranska County, Rijeka, Croatia; ^2^Department of Physiology, Immunology and Pathophysiology, University of Rijeka, Faculty of Medicine, Rijeka, Croatia; ^3^Department of Basic Medical Sciences, University of Rijeka, Faculty of Health Studies, Rijeka, Croatia; ^4^Department of Health Ecology, University of Rijeka, Faculty of Medicine, Rijeka, Croatia; ^5^Department of Internal Medicine, General Hospital Varaždin, Varaždin, Croatia; ^6^Community Health Centar of Istra County, Pula, Croatia; ^7^Vuk Vrhovac University Clinic for Diabetes, Endocrinology and Metabolic Diseases, Merkur University Hospital, Zagreb, Croatia; ^8^School of Medicine, University of Zagreb, Zagreb, Croatia; ^9^School of Medicine, Josip Juraj Strossmayer University of Osijek, Osijek, Croatia; ^10^Department of Endocrinology, Diabetes and Metabolic Diseases, Clinical Hospital Centre Rijeka, Rijeka, Croatia; ^11^Department of Internal Medicine, University of Rijeka, Faculty of Medicine, Rijeka, Croatia

## Abstract

**Background:**

Associated with epidemics of obesity, nonalcoholic fatty liver disease (NAFLD) is becoming the most prevalent liver disease worldwide. The cornerstone of therapy for NAFLD is lifestyle intervention, mainly focused on weight loss. Significant weight loss results from energy-restricted diets, regardless of macronutrient distribution. An anti-inflammatory diet was related to lower odds of NAFLD among daily alcohol drinkers and individuals with metabolic syndrome. This study aims to evaluate the effect of an energy-reduced anti-inflammatory diet on liver status in younger adults with obesity after a 6-month follow-up.

**Methods:**

A two-arm randomized controlled trial surveyed 81 participants' (mean age, 43 years) anthropometric and body composition changes. Metabolic status was determined with glycaemic and lipid status, inflammatory status with hs-CRP, IL-6, and TNF-*α*, and liver status with liver enzymes, NAFLD-FLS, FLI, and FIB-4 indices. The inflammatory potential of the diet was assessed by the Dietary Inflammatory Index, DII®.

**Results:**

Energy-restricted anti-inflammatory diet resulted in significant weight loss (−7.1%, *p* < 0.001), in reducing the visceral adiposity (−22.3%, *p* < 0.001), metabolic (HOMA-IR, −15.5%; total cholesterol, −5.3%; LDL-C, −4.6%; triglycerides, −12.2%), and inflammatory biomarkers (hs-CRP, −29.5%; IL-6, −18.2%; TNF-*α*, −34.2%), with significant improvement of liver parameters (NAFLD-FLS, −143.4%; FLI, −14.3%; FIB-4, −2.5%).

**Conclusion:**

The study showed the effectiveness of the anti-inflammatory diet with significant improvement of liver parameters in younger adults with obesity, which may reinforce the effectiveness of nutrition-based lifestyle programs, with an anti-inflammatory dietary approach for the treatment and resolution of NAFLD.

## 1. Introduction

In obesity, the accumulation of fat in the liver is associated with insulin resistance and subacute liver inflammation [1,2]. The most common subtype of liver fat accumulation is a nonalcoholic fatty liver disease (NAFLD), which progresses in individuals without excessive alcohol consumption, strong genetic predispositions, or use of steatogenic medication [3]. It was suggested that NAFLD is a risk factor for cardiovascular diseases and extrahepatic cancers, because NAFLD can potentially progress into nonalcoholic steatohepatitis and the later into cirrhosis and hepatocellular carcinoma [4]. Due to the obesity multisystem effect, the prevalence of NAFLD goes associated with the prevalence of obesity, making the most serious health threat responsible for increasing the number of cardiovascular, oncologic, and liver-related morbidity and mortality [5]. The burden of obesity-associated NAFLD can be ameliorated with lifestyle interventions, mainly by inducing weight loss and maintain a healthy body weight [6]. Short-term energy intake restriction resulted in a reduction in intrahepatic triglyceride storage [7,8], but the metabolic and hepatic effects of such lifestyle changes are less well understood [9]. To improve liver steatosis, 3%–5% loss in body weight is recommended, with greater liver status improvements when the weight loss is higher [10,11]. Marin-Alejandre et al. [12] showed that higher adherence to the Mediterranean diet resulted in a greater reduction in body weight, total fat mass, and hepatic fat and suggested additional benefits to weight loss in the treatment of obesity and associated comorbidities, such as NAFLD. However, the effects of dietary components, characteristics, and strategies for NAFLD treatment require more research [12–14]. The growing body of scientific evidence suggests that diet and dietary components are involved in the path of inflammation and consequently the pathogenesis of NAFLD. A diet with higher proinflammatory potential has been shown to be associated with higher odds for NAFLD development [15,16]. According to ATTICA study results, an anti-inflammatory diet was related to lower odds of NAFLD among daily alcohol drinkers and individuals with metabolic syndrome [17]. The PREDIMED substudy [16] reinforced the concept that obesity is associated with liver damage and revealed that the consumption of a proinflammatory dietary pattern might contribute to obesity and fatty liver disease features. The authors suggested that a well-designed precision diet containing acknowledged anti-inflammatory dietary components could specifically prevent and ameliorate obesity-related nonalcoholic fatty liver manifestations [16].

In this study, we present the changes in metabolic and hepatic parameters achieved with an energy-reduced anti-inflammatory diet among younger adults with obesity, with or without obesity-related complications.

## 2. Participants and Methods

### 2.1. Participants

The participants were recruited during their first visit to the obesity outpatient clinic at the Clinical Hospital Centre Rijeka, Croatia. The inclusion criteria were an age of 18 to 50 years, BMI ≥ 30 kg/m^2^ with or without obesity-related complications, and stable body weight for the previous three months. Exclusion criteria were cigarette smoking within 6 months before study initiation, chronic heart, kidney, and/or severe liver disease, malignant disease or history of malignant disease, use of anti-inflammatory or immunosuppressive drugs or medications for weight loss, changes in chronic medications, active infection or surgical procedure in the previous three months, food allergy or intolerance to any anti-inflammatory diet constituent, pregnancy, and lactation.

### 2.2. Study Protocol

**Figure 1:**
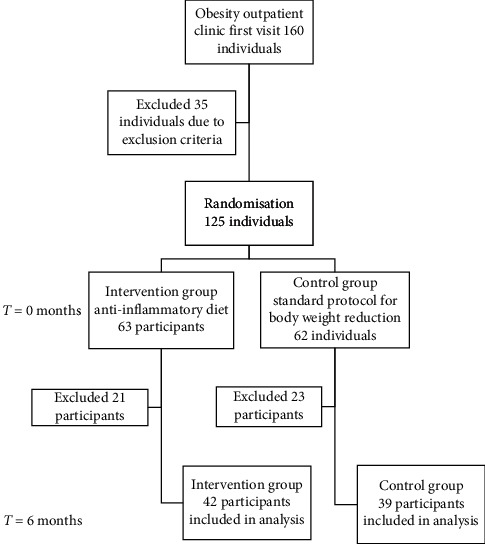
The flowchart of participants in the study trial.

This six-month two-arm randomized controlled trial was designed to compare the effects of two dietary plans for weight loss with different nutritional characteristics on body weight, body composition, and metabolic, hepatic, and inflammation statuses in young adults with obesity. After the study presentation and baseline assessments, the recruited participants were randomly assigned to the anti-inflammatory diet (AID) group or the control diet (CD) group using a web-based randomization system (https://www.random.org/), administrated by trained medical personnel not engaged in any other study procedure. The study was conducted between March and October 2019 at Clinical Hospital Centre Rijeka, Croatia, previously approved by the ethics committee of the Clinical Hospital Centre Rijeka (Reg. No: 2170-29-02/15-16-4, January 31st, 2017) and conducted in line with the Declaration of Helsinki. All of the participants provided written informed consent before participating in the study. The study protocol has been registered with clinicaltrials.gov: NCT03987776 and has been described in detail elsewhere [18]. After the randomization of the participants, a comprehensive assessment was carried out at the baseline and the endpoint of the study, including anthropometric measurements, body composition, biochemical, and dietary assessments. The questionnaire used in this study contained standard sociodemographic information, physical activity level, dietary habits, medications, dietary supplements use, and self-reported stress. Except for demographics, the questionnaire was repeated at the study end. The flowchart of the participants is shown in Figure 1.

### 2.3. Dietary Intervention

At the educational workshops, held each month by a clinical dietitian, the AID group participants were instructed and strongly encouraged to follow an energy-restricted diet, based on low glycaemic foods, whole-grain products, legumes, colourful vegetables and fruits, nuts, seeds, marine fish, olive oil, green/black tea, and multiple spices and herbs. The CD group participants were instructed and strongly encouraged to follow an isocaloric standard diet protocol for bodyweight reduction (55–60% carbohydrates, 25–30% fat, and 15–20% protein) [19]. Each dietary intervention has been described in a study protocol [18]. The AID group participant used more often olive oil, colourful low glycaemic index vegetables and fruits, nuts, seeds, onion, garlic, various spices, marine fish, and fermented dairy products and avoided red and processed meat and industrially processed foods to overcome an overlap in recommended daily intake of vegetables, fruits, legumes, whole grains, nuts, green tea, and herbs among the CD group. Daily resting energy expenditure was calculated for each participant according to Mifflin-St. Jeor's equation [20] using their baseline anthropometric measurements and then multiplied with the activity factor based on information from the physical activity questionnaire [21]. The value obtained from these equations was reduced by 25%, thus providing the recommended energy intake for each participant. The adjustments of the number and quantity of servings of each food group were made accordingly. At each workshop, meal planning with recipes, food serving sizes, specific food consumption, and personal goal-setting was discussed. Participants who had missed the educational workshop were provided with workshop materials.

The compliance with given dietary recommendations was monitored with 3-day food intake records (covering two weekdays and one weekend day) that each participant was asked to fulfil before a monthly group meeting (overall six 3-day food intake records). The dietary records results were discussed with each participant, and those whose dietary intervention adherence was less than 75% were considered as noncompliant and withdrawn from the trial. The baseline dietary habits that were assessed with a 133-item food frequency questionnaire (FFQ) [22] were discussed with each participant to correctly follow the dietary intervention. A Croatian food composition database [23] was used to calculate the energy and dietary components intake, and certain nutrients such as caffeine, *β*-carotene, omega-3, and omega-6 fatty acids intake were calculated using Danish [24] and American food composition database [25], the Phenol-Explorer 3.0 database [26], and USDA database [27]. The contents of the various polyphenols were multiplied by their retention factors, due to meal thermal processing [28].

The assessment of the inflammatory potential of the diet was done with the Dietary inflammatory index, DII^®^ [29], which included all of its 45 parameters. For DII® calculation, firstly we calculated a z-score by adjusting each participant's dietary intake data against a reference global daily mean and standard deviation (SD) intake for each parameter. The global dietary intake data were based on consumption data from 11 countries [29]. For decreasing the effect of right-skewing of the dietary data, the *z*-score was expressed as a proportion (i.e., with the value from 0 to 1). The centring of provided scores on 0 was achieved by doubling the proportion and subtracting 1. The resulting centred proportion score for each dietary parameter was multiplied by its respective parameter-specific inflammatory effect score and then each calculated 45 scores were summed to achieve an overall DII score of each participant [29]. The positive DII® score values specified a proinflammatory diet, and negative values an anti-inflammatory diet [29]. The dietary data were provided from the food frequency questionnaire (FFQ) [18] at the study baseline and its end, for obtaining the intake frequency (from once per month to a few times per day) and food and beverage portion size (small, medium, and large) information. To the standard list of 97 food items that were represented in the FFQ, for this trial, we added 36 food items and herbs and spices with anti-inflammatory properties.

### 2.4. Anthropometric, Body Composition, and Biochemical Assessment

The assessment of anthropometric measurements (body weight, height, and waist circumference), body composition by the bioelectrical impedance method (Seca mBCA 515, Seca gmbh and co. Kg, Hamburg, Germany), and blood pressure (Omron® HEM 705 CP, Health-care Co, Kyoto, Japan) was carried out under fasting conditions at the obesity outpatient clinic at the Clinical Hospital Centre Rijeka, Croatia following standardized procedures, as previously described [18]. Body Mass Index (BMI) was calculated as the bodyweight divided by the squared height (kg/m^2^). Biochemical assessments, including concentrations of blood glucose, glycated haemoglobin (HbA1c), aspartate aminotransferase (AST), alanine aminotransferase (ALT), gamma-glutamyl transferase (GGT), total cholesterol (TC), high-density lipoprotein cholesterol (HDL-C), low-density lipoprotein cholesterol (LDL-C), triglyceride (TG), and high sensitivity C-reactive protein (hs-CRP) were measured on an Olympus 5800 (Olympus) with specific commercial kits. Insulin was analysed with the CLIA method on Immulite 2000xp, Siemens. The ELISA method was used for the measurement of interleukin-6 (IL-6) and tumor necrosis factor-alpha (TNF-*α*) with assay kits purchased from eBioscience™ (Thermo Fisher Scientific, Waltham, USA).

The insulin resistance was assessed using the Homeostasis Model Assessment Index (HOMA-IR) [30]. The metabolic syndrome was assessed by the presence of three or more parameters according to the definition by the International Diabetes Federation Task Force on Epidemiology and Prevention [31].

Currently, the “golden” standard for NAFLD diagnosis is liver biopsy, but it is invasive, in some cases clinically unavailable, also time and money consuming. The use of blood biomarkers and particular indices for NAFLD diagnosis may be useful to select individuals who need NAFLD ultrasonography screening as a noninvasive tool for assessing fibrosis and making the decision of whether to perform a liver biopsy. It was shown that the vast majority of patients will never develop severe liver disease, so it is neither realistic nor necessary to perform a liver biopsy in all patients [5]. Therefore, for estimation of liver fat content in NAFLD, i.e., hepatic steatosis, we used NAFLD-FLS score and modified Fatty Liver Index, and for estimating the liver fibrosis possibility, we used Fibrosis Index based on four factors (FIB-4 index).

NAFLD-FLS score [32,33] was assessed according to the formula: NAFLD-FLS = – 2.89 + 1.18 ×  MetS (yes: 1; no: 0) + 0.45 × diabetes mellitus (yes: 2; no: 0) + 0.15 × insulin (mU/L) + 0.04 × AST (U/L) – 0.94 × AST/ALT. We used a NAFLD-FLS cutoff of > –0.64 to classify those with hepatic steatosis.

A modified Fatty Liver Index (FLI) [34,35] was assessed according to the formula: liver fat (%) = 10(−0.805 + 0.282 *∗* metabolic syndrome (yes = 1; no = 0) + 0.078 *∗* type 2 diabetes (yes = 1; no = 0) + 0.525 LOG(fS−insulin (mU/L) + 0.521 *∗* LOG(fS−AST (U/L) − 0.454 *∗* LOG (AST/ALT), with a cutoff of >0.8 for classifying those with hepatic steatosis.

FIB-4 index [36] was assessed according to the following formula: FIB-4 = (age × AST)/[PLT(×10^9^/L) × (√ALT)], with a cut off of >1.45 for classifying the possibility of liver fibrosis.

### 2.5. Statistical Analyses

The statistically significant sample size for this study was estimated using the data from a recent randomized controlled trial that compared the effects of two dietary strategies for weight loss with different nutritional characteristics among subjects with obesity and NAFLD [12]. With a 95% confidence interval (*α* = 0.05) and a statistical power of 90% (*β* = 0.9), group size ratio 1 : 1, and using *t* test for repeated measures, it was calculated that 42 participants per group were needed, but considering the estimated dropout rate of 25%, at least 53 participants per each study group were considered for the study inclusion.

The mean value (standard deviation) described the studied variables. The evaluated variables were tested for normality of the distribution by the Kolmogorov-Smirnov test. The differences between the study groups were compared with Student's *t* test or the Mann–Whitney *U* test when appropriate. The differences between the beginning and the end of the intervention period within each group were analysed by a paired Student's *t* test or Wilcoxon test when appropriate. Categorical variables were compared using a chi-squared test. All parameters' changes were calculated with *z*-score ((mean after intervention-baseline mean)/baseline mean ×100). Linear regression analyses were used to evaluate the potential association between the anthropometry, body composition, metabolic, inflammatory, and hepatic status variables with the inflammatory potential of the diet, with adjustments for age, sex, educational level, physical activity, and obesity degree. All tests were performed with Statistica 12.7 for Windows (Statsoft Inc, Tulsa, OK, SAD), which were regarded as 2-tailed, and *p* values <0.05 were considered as statistically significant.

## 3. Results

After 6 months of nutritional intervention, out of 125 participants fulfilling inclusion and exclusion criteria, 63 were randomized to the AID group and 62 to the CD group. A total of 81 participants (42 in the AID group and 39 in the CD group) completed the trial evaluation and entered in all trial calculations. Noncompliance with the dietary recommendations was the main reason for exclusion with dropouts that were similar for both groups. The flowchart of participants has been shown in Figure 1. The majority of trial participants in both groups were female (93%, *p* < 0.001 vs 90%, *p* < 0.001) (Table 1). There were almost half of the participants with three or more components of the metabolic syndrome in the AID group. At the end of the trial, the proportion of participants fulfilling the criteria for metabolic syndrome decreased by almost half in the AID group (*p* = 0.042) and by 30% in the CD group (*p* = 0.314). The number of participants with hyperglycaemia as assessed with HbA1c values higher than 6.5 reduced in half after the 6 months of the trial in the AID group, but not significantly (*p* = 0.057), while it significantly reduced in the CD group (*p* = 0.003). In the AID group, hepatic steatosis assessed with NAFLD-FLS and with FLI was detected in 43% and 48% of participants, respectively, and both reduced in half at the trial end, but not significantly. In the CD group, hepatic steatosis assessed with NAFLD-FLS was detected at 38% of participants. That proportion reduced significantly for third (p = 0.019), and while assessed with FLI, it reduced by 14%, but not significantly. . The possibility for liver fibrosis had around 5% of participants in both dietary groups and significantly reduced to 0% at the trial end *p* < 0.001 and *p* < 0.001, respectively).

A significant weight loss has been achieved in both dietary groups (−7.1%, *p* < 0.001 vs −6.2%, *p* < 0.001) (Table 1). Also, BMI, total body fat, and visceral fat decreased significantly in both dietary groups, while the proportion of nonfat tissue significantly increased. No statistically significant differences were found between the intervention groups for the mentioned variables nor at the baseline nor the trial end (Table 1).

Both dietary groups showed improvements in glycaemic, lipid, and inflammatory parameters. Fasting glucose, insulin, and HOMA-IR were reduced in both groups; however, these changes were statistically significant only in the CD group (Table 1). Moreover, the CD group achieved significant reductions in the total and LDL-cholesterol concentrations (*p* = 0.002 and *p* < 0.001, respectively) (Table 1). Biomarkers of inflammation were significantly reduced in both groups. The AID group participants achieved greater reduction in TNF-*α* (−34.2%, *p* = 0.002 vs −10.5%, *p* = 0.001, respectively), while the CD group participants reduced hs-CRP (29.5%, *p* = 0.003 vs −42.12%, *p* = 0.010, respectively) and IL-6 concentrations (−18.2%, *p* = 0.013 vs −26.9%, *p* = 0.002, respectively) slightly more than the AID group participants. Only the changes in glycaemic parameters (*p* = 0.001, *p* = 0.050, *p* = 0.048, *p* = 0.040, respectively), IL-6 (*p* = 0.001), and TNF-*α* (*p* = 0.001) from the baseline to 6 months of intervention differed significantly between the dietary groups.

A reduction in liver enzymes (AST, ALT, and GGT) was observed in both groups; however, these changes were statistically significant only for GGT (−21.3%, *p* = 0.011 in the AID group and −14.3%, *p* = 0.003 in the CD group) (Table 2). A significant reduction in the Fatty Liver Index was achieved with both dietary interventions (*p* = 0.040 and *p* = 0.006, respectively). NAFLD-FLS and FIB-4 indices notably reduced in both groups but not significantly (Table 2). Only the changes in GGT (*p* = 0.040) and FLI (*p* = 0.047) from the baseline to 6 months of intervention differed statistically significant between the two dietary strategies.

Furthermore, there were no significant differences at baseline concerning dietary intake, except for higher intake of MUFA (*p* = 0.018), omega-3 fatty acids (*p* = 0.010), total polyphenols (*p* = 0.002), and a lower intake of dietary cholesterol (*p* = 0.004) by the AID group (Table 3). Regarding changes from baseline to 6 months of intervention, both dietary groups achieved a significant reduction in energy intake (*p* < 0.001) and saturated fat energy share (*p* < 0.001). Both groups significantly raised the proportion of total energy intake from proteins (*p* < 0.001) and MUFA (*p* < 0.001), intake of fibre (*p* < 0.001), and total polyphenols (*p* = 0.019 and *p* < 0.001, respectively). The AID group significantly reduced the proportion of total energy intake from carbohydrates (*p* < 0.001) and dietary cholesterol intake (*p* = 0.030) and significantly increased the proportion of total energy intake from total fat (*p* = 0.021), PUFA (*p* = 0.029), and omega-3 fatty acids (*p* < 0.001). The CD group significantly reduced the proportion of total energy intake from alcohol (*p* = 0.037). Both dietary groups significantly raised the intake of flavones (*p* < 0.001 and *p* = 0.037, respectively) and reduced the intake of flavonones (*p* < 0.001, *p* < 0.001, respectively), which the CD group reduced significantly more than AID group (*p* = 0.002, *p* < 0.001, respectively). The intakes of other flavonoid subgroups were raised by both dietary groups but not significantly. As expected, the AID group significantly decreased the DII® value (*p* = 0.002), significantly more than the CD group (*p* < 0.001).

Linear regression analyses (adjusted by a group of intervention, age, sex, physical activity, medication use, and obesity comorbidities) were performed to evaluate the anthropometric, biochemical, and dietary factors potentially involved with liver parameters after the 6 months of the dietary intervention (Tables 4 and 5). Models were not adjusted for total dietary energy and dietary supplements intake because they are the DII^®^ components. We noticed that the weight loss and reduction of BMI and visceral fat tissue were associated with improvements in hepatic status but not significantly (Tables 4 and 5). The decrease in total fat tissue was significantly associated with a reduction in Fatty Liver Index (*p* = 0.037) and Fibrosis Index based on four factors in the CD group after adjustment (*p* = 0.021) (Table 5). Regarding inflammatory markers, we found that their reduction was associated with improvements in hepatic status, but not significantly, except for IL-6 with FLI in the CD group after adjustments (*p* = 0.020). Concerning dietary factors, the decrease of DII® and energy was significantly associated with the decrease of FIB-4 index in the AID group (*p* = 0.044 and *p* = 0.042, respectively). Also, in the same dietary group, the increase in total dietary fat influenced the FIB-4 index increase after the adjustment (*p* = 0.031). At the same time, we found that, among the AID group, the decrease of flavones and flavonones was associated with improvement in FIB-4 (*p* = 0.043 and *p* = 0.047, respectively) and of flavonols with FLI (*p* = 0.048) after adjustment (Table 4). After adjustment, it was found that the decrease in flavones and in anthocyanidins resulted in significant improvements of FLI in the CD group (*p* = 0.027 and *p* = 0.012, respectively). The increase in protein intake resulted in improvements in FLI (*p* = 0.043) among CD group participants after adjustment (Table 5).

## 4. Discussion

The present randomized controlled trial that compared the effects of two energy-restricted dietary interventions on anthropometry, body composition, and biochemical parameters and the non-invasive parameters of liver status in younger adults with obesity resulted in noteworthy improvements in liver enzymes, and in hepatic status indices. Both dietary groups achieved significant improvements in their anthropometric and body composition parameters, with no significant difference between them after the 6 months of the trial. Participants that consumed an energy-reduced anti-inflammatory diet achieved a greater reduction in total body weight, while participants in the CD group obtained slightly larger reduction of total fat tissue and visceral adipose tissue associated with improvement in FLI and FIB-4. The AID group achieved a more significant reduction of GGT and similar of FLI. An important contributing factor to adverse clinical outcomes, including NAFLD's pathophysiology, is excess body weight [37]. For that reason, weight loss management had been suggested as the most important factor for NAFLD treatment [37,38].

It was shown that weight loss of ≥3% was able to improve liver steatosis, although at least 5% weight loss is needed to improve inflammation and hepatic histology [39] and to stabilize fibrosis [40,41]. Besides, the weight loss of 7% or more resulted in improvement of nonalcoholic steatohepatitis (NASH) in 65–90% of patients [40–42]. In our study, both studied groups reached on average 7% loss of their baseline weight and achieved a significant reduction in the Fatty Liver Index and GGT level. A higher decrease in total adipose tissue was observed in the CD group which was significantly associated with lower liver fibrosis estimated with FIB-4 index. On the other hand, it was noticed that visceral adipose tissue reduction in the CD group was associated with improvements in liver steatosis and liver fibrosis after adjustments for potential confounders such as age, sex, physical activity, use of medications, and obesity comorbidities. The CD group had higher FIB-4 values at the baseline which perhaps did not notably reduce after 6 months of the trial in those who had a lower reduction of total and visceral adipose tissue, but after the adjustments, the reduction of total fat tissue was significantly associated with its reduction. The reduction of total fat tissue among participants in the AID group has been associated with improvements in the NAFLD-FLS and FLI index and in liver fibrosis estimated with FIB-4 index but not significantly. It was shown that liver fibrosis progression does not occur in all patients with diagnosed NAFLD and not at the same rate [5], which is in line with the results of our study.

The distribution of body fat is a main pathophysiological mechanism for metabolic disease, where abdominal obesity differs from a more equally fat distribution [43]. Free fatty acids (FFAs) released from hypertrophic adipocytes, especially from visceral adipose tissue, induce systemic and hepatic insulin resistance which successively intensifies the release of FFAs from adipose tissue. Excessive amounts of circulating FFAs ultimately lead to hypertriglyceridemia and consequently NAFLD [44]. Furthermore, the accumulation of liver fat is strongly associated with diminishing adipose tissue insulin sensitivity [45]. NAFLD appears to increase the chances of developing nonfatal coronary heart disease, ischemic stroke, or cardiovascular death by more than 50% in patients with T2D [43]. In our study, 37% of participants had HbA1c ≥ 6.5% at baseline, indicating the diagnosis of type 2 diabetes. After the dietary intervention, this number decreased to only 14% of participants suggesting better glycaemic control or even diabetes remission in those not taking or eliminating diabetes medications. Although all participants in this trial improved their glycaemic status, the CD group participants significantly more improved their insulin resistance assessed with HOMA-IR. HOMA-IR in the AID group was slightly higher at baseline, with a larger values array, which can be a cause one of the reasons for an insignificant decrease in HOMA-IR values. NAFLD consists of two clinicopathological entities: a simple steatosis and NASH. Simple steatosis is detected as lipid accumulation in hepatocytes with little or no inflammation and fibrosis, while NASH comprises inflammation and fibrosis [46,47]. During the adipose tissue expansion, the modification of secreted adipokines towards a more steatogenic, inflammatory, and fibrogenic profile results with a higher production of cytokines. The excess of proinflammatory cytokines, and at the same time, a deficiency of anti-inflammatory cytokines has been observed in the progression of NASH in the liver and visceral adipose tissue [48]. With this trial, the biomarkers of inflammation were significantly reduced in both dietary groups. The AID group participants achieved greater reduction in TNF-*α*, and the CD group in IL-6. The reduction of hs-CRP was associated with the improvements in liver status in both dietary groups, and in the CD group, the reduction of IL-6 was associated with improvements of FLI. The significant reduction of inflammatory markers in both dietary groups can be explained by significant weight loss and by reduction of total and visceral adipose tissue, which is supported by the suggestion that the weight loss has a central role in reducing the inflammatory makers [49]. Additionally, it has been showed that, independent of the diet's composition, a hypocaloric diet had an anti-inflammatory effect [49], and by that, it may represent the most effective treatment for metabolic disorders by an effect on reducing the visceral adiposity, and the incidence of T2D, and the inflammation [49]. Both dietary groups in this study significantly and in similar quantities reduced their energy intake. However, the CD group slightly more, because they had higher energy intake at the baseline. Still, the reduction of energy intake by the AID group participants was significantly associated with improvements in FIB-4. A recent randomized controlled study examining two dietary strategies in subjects with obesity and NAFLD showed that the effect of weight loss in inflammatory markers might be greater when supplemented by a higher intake of fruits and vegetables [12]. The authors showed that the greater effect was achieved by a diet with high adherence to the Mediterranean diet [12]. In this trial, both dietary strategies had characteristics of the Mediterranean diet, thus overlapping in certain recommendations. Therefore, a higher intake of foods with anti-inflammatory characteristic was more promoted among the AID group participants, which is in detail described elsewhere [18]. Our study results are in line with the conclusion from a recent review and meta-analysis that a higher intake of fruits and vegetables leads to a reduction in proinflammatory mediators [50]. Fruits and vegetables abound with natural compounds that are found to be effective in the alleviation of NAFLD and its related comorbidities [51]. Specifically, these are flavonoids, which showed their protective effects in all stages of NAFLD prevention, development, complications, and consequences [51], although mostly observed in animal models, with experimentally induced hepatic steatosis and with higher doses that could be achieved with the usual diet. Each flavonoid, regardless of their diet sources, has its potential and biological effects, and a synergistic effect may be realised if these flavonoids are consumed together [51]. Furthermore, it was suggested that flavonoids may decrease body weight and fat deposition in visceral tissues and the liver, partly by increasing fatty acid *β*-oxidation and suppressing lipogenesis [51]. In this study, we showed total polyphenols and various flavonoids subgroups intake in both dietary groups, and their intake changes with the study trial.

After the period of 6 months of the intervention, the intake of total polyphenols and flavonoids increased in both dietary groups but significantly more in the CD group which could explain the improvement in the liver enzymes and NAFLD-FLS seen in this group. Although the AID group had significantly higher intake of flavonoids subgroups than the CD group, their intake was significantly associated with improvements in FLI and FIB-4 after the adjustments for potential confounders but not in NAFLD-FLS, which is seen in the CD group. The lack of significant associations with the intake of total polyphenols and flavonoids subgroups in the AID group could be explained by their relatively high intake at the baseline, compared with the CD group. There is still limited evidence on the association of polyphenols and specific flavonoids subgroups to NAFLD, NASH, and liver fibrosis, so this study results provide valuable information regarding this issue, particularly on the relationship between inflammatory markers and dietary strategies in the treatment of NAFLD.

There are some limitations and strengths in this study that should be recognized. This study included participants with obesity, with and without obesity-related complications, which includes NAFLD. NAFLD was evaluated using noninvasive techniques instead of a liver biopsy, which is currently the most reliable method for detecting steatohepatitis and fibrosis, specifically in subjects with NAFLD. In this study, adults with obesity participated, so the intent was to use noninvasive and rather fast parameters for NAFLD detection. Liver biopsy is a procedure that is limited by its cost and sampling errors and also with procedure-related complications [10], so we used scoring systems that need to be validated. However, we carried out a solid evaluation of liver status, and the design of this study was well protocoled regarding its procedure, methods used, specifically dietary methods that were clarified by the dietitian, and monitored for diet adherence, which resulted in the relatively low exclusion of the participants. Also, the concerns about monitoring adherence and sustainability of dietary intervention are overcome by frequent cooperation with the participants and by reviewing their 3-day food diary once per month in each dietary group according to dietary recommendations. The participants in both dietary groups had baseline average diet with slight anti-inflammatory potential, which all increased during the trial, the AID group participants significantly as expected. That increase of anti-inflammatory potential in the AID group was only significantly associated with improvements in liver fibrosis status, after the adjustments for confounders. Potential confounders that significantly reduced were obesity comorbidities such as metabolic syndrome components in the AID group. Furthermore, all participants in this trial were individuals with obesity, and although they all significantly reduced their baseline weight and adipose tissue, most of them remained in the obesity class after the 6 months of the trial. All of the above may be the reason that there were no observed significant associations of dietary change towards a more anti-inflammatory diet with improvements in liver status. Still, the observed alleviation of obesity comorbidities, including liver status, indicates their possible significant improvements if they continue with given anti-inflammatory dietary recommendations. Another important fact to point out is that our study participants were individuals with obesity younger than 50 years, meaning that among them, there were a specific number of individuals with so-called “metabolically healthy obesity,” which is more often observed in young, physically active individuals, with better nutritional status and low levels of ectopic and visceral fat storage and not showing metabolic abnormalities, such as insulin resistance [52]. In addition to that, by detecting any liver parameters normality deviation and/or diagnosis of NAFLD in individuals with obesity at their younger age could prevent health complications in the future, along with reducing the costs of the medical treatments. To the present, it is still difficult to single out the effective diet or nutrient/s regarding NAFLD treatment; yet, the study results showed improvements in hepatic parameters associated with weight loss, reduction of total and visceral adipose tissue, and changes in energy and nutrients intake, specifically in flavonoid subgroup intake.

## 5. Conclusions

The study results showed the effectiveness of the anti-inflammatory diet in weight loss, in reducing the visceral adiposity and metabolic and inflammatory biomarkers and significant improvement of hepatic parameters in younger adults with obesity. Since there are still limited data about the specific dietary approach for ameliorating the NAFLD pathophysiology, the presented results may reinforce the effectiveness of nutrition-based lifestyle programs, with diet such as an anti-inflammatory dietary approach for the treatment and resolution of NAFLD.

## Figures and Tables

**Table 1:** Patient characteristics and changes in anthropometric and biochemical parameters at baseline and after 6 months of dietary intervention.

Variable	Anti-inflammatory diet group (*n* = 42)				Control diet group (*n* = 39)				Baseline *p*-value^b^	6-months *p*-Value^c^
	Baseline	6 months	Change (%)	*p*-value^a^	Baseline	6 months	Change (%)	*p*-value^a^		
Sex (men/women)	3/39		−	<0.001^d^	4/35		−	<0.001^d^	0.619^d^	−
Metabolic syndrome (yes)	20	11	−45.0	0.042^d^	13	9	−30.8	0.314^d^	0.191^d^	0.746^d^
*HbA1c* *≥* *6.5%*	12	5	−58.3	0.057^d^	18	6	−66.7	0.003^d^	0.102^d^	0.648^d^
*NAFLD-FLS* *>* *–0.64*	18	13	−27.8	0.062^d^	15	10	−33.3	0.019^d^	0.916^d^	0.596^d^
*FLI* *>* *0.8*	20	15	−25.0	0.184^d^	21	18	−14.3	0.749^d^	0.575^d^	0.339^d^
*FIB-4* *>* *1.45*	2	0	−100.0	<0.001^d^	2	0	−100.0	<0.001^d^	0.938^d^	0.992^d^
Age (years)	43.6 (5.8)		−	−	41.7 (6.7)		−	−	0.178	−

*Anthropometry and body composition*										
Body weight (kg)	102.9 (14.2)	95.7 (11.7)	−7.1	<0.001	101.4 (21.9)	95.1 (21.4)	−6.2	<0.001	0.770	0.903
Body Mass Index (kg/m^2^)	35.4 (4.3)	32.9 (3.9)	−7.0	<0.001	33.4 (5.5)	31.0 (4.3)	−7.2	<0.001	0.179	0.119
Waist circumference (cm)	108.4 (8.4)	102.9 (7.8)	−5.1	<0.001	107.9 (10.1)	100.9 (10.0)	−6.5	<0.001	0.482	0.442
Total fat tissue (%)	44.9 (4.4)	42.3 (4.8)	−5.6	<0.001	45.6 (2.6)	42.2 (3.0)	−7.4	<0.001	0.505	0.755
Visceral adipose tissue (*l*)	3.1 (1.3)	2.4 (1.0)	−22.3	<0.001	3.5 (1.6)	2.6 (1.4)	−25.4	<0.001	0.376	0.798
Nonfat tissue (%)	55.1 (4.4)	57.8 (4.7)	4.8	<0.001	54.4 (2.6)	57.2 (2.4)	5.2	<0.001	0.484	0.587
Skeletal muscle tissue (kg)	27.4 (3.9)	26.2 (3.3)	−4.3	0.022	27.0 (6.2)	25.8 (7.3)	−4.4	0.005	0.449	0.085

*Biochemical parameters*										
Glucose (mmol/l)	5.7 (1.4)	5.5 (0.6)	−3.7	0.284	5.6 (0.5)	4.9 (0.6)	−13.1	<0.001	0.107	0.001
HbA1c (mmol/mol)	35.3 (6.5)	34.7 (7.6)	−1.7	0.855	38.3 (4.9)	38.4 (4.7)	0.1	0.121	0.128	0.050
Insulin (mU/l)	18.2 (11.7)	16.2 (10.0)	−11.1	0.946	16.1 (4.9)	11.7 (3.9)	−27.1	0.008	0.419	0.048
HOMA-IR (pmol/l)	4.8 (3.9)	4.1 (3.0)	−15.5	0.307	4.0 (1.3)	2.5 (0.9)	−36.3	0.002	0.572	0.040
Total cholesterol (mmol/l)	5.3 (1.1)	5.0 (1.34)	−5.3	0.594	5.8 (0.7)	5.4 (0.8)	−7.7	0.002	0.028	0.193
HDL-C (mmol/l)	1.4 (0.2)	1.5 (0.53)	10.2	0.058	1.3 (0.2)	1.3 (0.1)	−0.8	0.073	0.642	0.127
LDL-C (mmol/l)	3.3 (1.0)	3.2 (0.99)	−4.6	0.354	3.8 (0.6	3.4 (0.6)	−12.0	<0.001	0.031	0.343
Triglycerides (mmol/l)	1.3 (0.9)	1.2(0.56)	−12.2	0.445	1.5 (0.4)	1.3 (0.5)	−11.3	0.393	0.008	0.144
Platelet (×10^9^/l)	261.3 (73.4)	248.3 (77.1)	−5.0	0.289	290.9 (104.3)	286.9 (85.3)	−1.4	0.226	0.268	0.049
hs-CRP (mg/l)	6.3 (5.5)	4.4 (4.29)	−29.5	0.003	6.8 (4.1)	3.9 (0.9)	−42.2	0.010	0.311	0.662
IL-6 (pg/mL)	0.8 (0.6)	0.6 (0.36)	−18.2	0.013	1.3 (0.9)	1.0 (0.8)	−26.9	0.002	<0.001	0.001
TNF-*α* (pg/mL)	0.4 (0.2)	0.3 (0.09)	−34.2	0.002	1.7 (0.3)	1.5 (0.4)	−10.5	<0.001	0.001	<0.001

Data are presented as number or the mean (SD). NAFLD-FLS, Nonalcoholic Fatty Liver Disease Liver Fat Score; FLI, Fatty Liver Index; FIB-4, Fibrosis Index based on four factors; HbA1c, glycated haemoglobin; HOMA-IR, Homeostatic Model Assessment for Insulin Resistance; HDL-C, high-density lipoprotein; LDL-C, low-density lipoprotein; hs-CRP: high sensitivity C-reactive protein; IL-6, interleukin-6; TNF-*α*, tumor necrosis factor-alpha. ^a^Comparison within dietary groups (baseline and after 6 months). ^b^Baseline differences between the AID and CD groups. ^c^Differences after 6 months between the AID and CD groups. ^d^Chi-squared test for baseline differences between the AID and CD groups.

**Table 2:** Liver parameters at baseline and after 6 months of dietary intervention.

Variable	Anti-inflammatory diet group (*n* = 42)				Control diet group (*n* = 39)				Baseline *p* value^b^	6 months *p* value^c^
	Baseline	6 months	Change (%)	*p* value^a^	Baseline	6 months	Change (%)	*p* value^a^		
AST (IU/L)	21.7 (7.9)	20.7 (6.1)	−4.8	0.516	24.0 (6.5)	23.0 (6.4)	−4.2	0.885	0.263	0.075
ALT (IU/L)	24.3 (13.5)	22.6 (11.2)	−6.8	0.914	31.9 (13.1)	29.3 (14.7)	−8.1	0.416	0.540	0.099
GGT (IU/L)	22.43 (9.9)	17.7 (6.7)	−21.3	0.011	25.4 (5.6)	21.8 (6.6)	−14.3	0.003	0.212	0.040
NAFLD-FLS	0.46 (2.2)	−0.2 (2.1)	−143.4	0.158	0.0 (0.8)	−0.1 (1.4)	−275.0	0.590	0.647	0.875
FLI	1.4 (0.6)	1.2 (0.5)	−14.3	0.040	1.6 (0.7)	1.3 (0.7)	−18.8	0.006	0.331	0.047
FIB-4	0.8 (0.2)	0.8 (0.2)	−2.5	0.452	1.2 (2.0)	0.7 (0.3)	−41.7	0.207	0.418	0.495

Data are presented as the mean (SD). AST, aspartate aminotransferase; ALT, alanine aminotransferase; GGT, gamma-glutamyl transferase; NAFLD-FLS, Nonalcoholic Fatty Liver Disease Liver Fat Score; FLI, Fatty Liver Index; FIB-4, Fibrosis Index Based On Four Factors. ^a^Comparison within dietary groups (baseline and after 6 months). ^b^Baseline differences between the AID and CD groups. ^c^Differences after 6 months between the AID and CD groups.

**Table 3:** Dietary intake at baseline and after 6 months of dietary intervention.

Variable	Anti-inflammatory diet group (*n* = 42)				Control diet group (*n* = 39)				Baseline *p* value^b^	6-months *p* value^c^
	Baseline	6 months	Change (%)	*p* value^a^	Baseline	6 months	Change (%)	*p* value^a^		
Energy (MJ)	10.0 (2.6)	6.9 (0.5)	−31.0	<0.001	11.2 (2.6)	7.6 (0.4)	−31.9	<0.001	0.129	<0.001
Protein (%MJ)	17.2 (1.7)	20.6 (2.6)	20.2	<0.001	17.1 (2.0)	21.3 (1.9)	25.0	<0.001	0.872	0.292
Carbohydrate (%MJ)	38.6 (6.1)	35.3 (7.7)	−8.6	<0.001	41.6 (4.7)	38.0 (3.7)	−8.7	0.535	0.063	0.131
Total fat (%MJ)	42.6 (6.5)	44.0 (6.2)	3.2	0.021	40.3 (3.7)	39.8 (3.7)	−1.3	0.005	0.133	0.006
MUFA (%MJ)	17.3 (4.5)	21.4 (6.9)	26.8	<0.001	14.9 (1.8)	16.0 (3.5)	8.3	0.856	0.018	<0.001
PUFA (%MJ)	7.3 (2.1)	8.4 (2.9)	17.4	0.029	7.1 (0.8)	6.5 (1.2)	−7.8	0.001	0.677	<0.001
Omega-3 (%MJ)	0.5 (0.3)	0.7 (0.4)	56.8	<0.001	0.3 (0.1)	0.3 (0.1)	0.0	0.109	0.010	<0.001
Omega-6 (%MJ)	0.3 (0.1)	0.3 (0.2)	−3.7	0.003	0.3 (0.1)	0.3 (0.1)	−3.2	0.159	0.210	0.071
Saturated fat (%MJ)	15.6 (2.9)	11.0 (24.6)	−29.5	<0.001	16.6 (2.5)	14.1 (33.8)	−14.8	<0.001	0.199	<0.001
Trans fat (%MJ)	0.7 (0.3)	0.7 (0.5)	14.3	0.279	0.7 (0.2)	0.7 (0.3)	−9.7	0.214	0.363	0.363
Cholesterol (mg)	380.8 (160.4)	318.5 (175.6)	−16.4	0.030	477.6 (463.0)	463.0 (125.6)	−3.0	0.643	0.004	<0.001
Fiber (g)	27.4 (11.3)	33.9 (5.2)	23.5	0.002	25.5 (6.7)	28.7 (3.9)	12.7	0.146	0.467	<0.001
Alcohol (%MJ)	1.6 (2.8)	1.6 (2.8)	0.0	0.999	1.1 (1.3)	0.9 (1.3)	−12.7	0.006	0.464	0.037
Total polyphenols (mg)	688.4 (240.0)	733.7 (106.0)	6.6	0.019	472.7 (200.8)	740.6 (98.3)	56.7	<0.001	0.002	0.817
Flavan-3-ol (mg)	28.8 (26.8)	15.8 (10.4)	−45.1	0.056^b^	23.4 (12.8)	8.3 (2.4)	−64.3	<0.001^a^	0.807^b^	<0.001^b^
Flavones (mg)	2.9 (2.03)	5.6 (3.4)	92.4	<0.001^a^	2.3 (1.4)	3.1 (1.5)	38.3	0.037^b^	0.366^b^	0.002^a^
Flavonols (mg)	147.4 (78.6)	149.4 (6.8)	1.4	0.856^a^	107.6 (34.8)	74.2 (40.0)	−31.0	<0.001^a^	0.029^a^	<0.001^a^
Flavonones (mg)	46.1 (37.9)	23.0 (22.9)	−50.2	<0.001^a^	65.6 (74.7)	3.6 (4.0)	−94.5	<0.001^a^	0.856^b^	<0.001^b^
Anthocyanidins (mg)	24.3 (34.6)	30.0 (28.4)	23.6	0.406^a^	15.5 (9.3)	24.3 (19.4)	56.4	0.606^a^	0.873^b^	0.426^a^
Isoflavones (mg)	0.4 (0.2)	0.6 (0.3)	55.6	0.864^a^	0.3 (0.4)	0.6 (0.5)	129.6	0.787^a^	0.816^a^	0.374^a^
DII®	−0.5 (2.3)	−2.0 (1.0)	283.0	0.002	−0.2 (1.3)	−0.3 (1.0)	30.4	0.725	0.579	<0.001

Data are presented as the mean (SD). DII®, Dietary Inflammatory Index. MUFA, monounsaturated fatty acids; PUFA, polyunsaturated fatty acids. ^a^Comparison within dietary groups (baseline and after 6 months). ^b^Baseline differences between the AID and CD groups. ^c^Differences after 6 months between the AID and CD groups.

**Table 4:** Regression analyses of the liver parameters after 6 months of dietary intervention as dependent variables and changes in anthropometric, biochemical, and dietary factors as independent variables in the AID group.^a^

Variable changes (Δ)	Anti-inflammatory diet group											
	NAFLD-FLS				FLI				FIB-4			
	Unadjusted		Adjusted		Unadjusted		Adjusted		Unadjusted		Adjusted	
	*β*	*p* value	*β*	*p* value	*β*	*p* value	*β*	*p* value	*β*	*p* value	*β*	*p* value
Bodyweight (kg)	−0.47	0.418	−1.00	0.280	−0.43	0.379	0.75	0.102	−0.70	0.381	−1.41	0.089
BMI (kg/m^2^)	−0.49	0.739	−1.95	0.291	−0.79	0.530	−1.44	0.111	−1.75	0.401	−1.59	0.222
Fat tissue (%)	−0.39	0.098	−0.21	0.282	−0.09	0.582	−0.09	0.265	−0.11	0.672	−0.35	0.070
Visceral adipose tissue (l)	−0.69	0.300	−0.53	0.324	−0.35	0.511	−0.21	0.308	−0.56	0.517	−0.80	0.097
hs-CRP (mg/l)	−0.16	0.120	−0.06	0.489	0.01	0.849	0.06	0.161	−0.18	0.192	−0.01	0.884
IL-6 (pg/mL)	0.15	0.985	−8.06	0.318	−5.14	0.462	−0.44	0.867	−1.71	0.876	−14.21	0.073
TNF-*α* (pg/mL)	−2.32	0.546	−9.16	0.131	−1.40	0.660	−2.68	0.199	−1.10	0.829	−9.68	0.059
DII®	−0.47	0.131	−1.28	0.225	−0.07	0.770	−1.10	0.060	−0.50	0.215	−2.27	0.044
Energy (MJ)	0.01	0.392	−0.01	0.204	−0.39	0.706	−0.01	0.067	0.01	0.436	−0.01	0.042
Proteins (%MJ)	−0.01	0.961	−0.21	0.198	−0.01	0.937	−0.11	0.118	0.10	0.522	−0.22	0.094
Total fat (%MJ)	−0.10	0.233	0.25	0.233	−0.05	0.467	0.19	0.081	0.01	0.930	0.55	0.031
Omega-3 (%MJ)	0.31	0.235	−0.32	0.394	0.16	0.458	−0.26	0.154	0.29	0.393	−0.69	0.074
Total polyphenols (mg)	−0.26	0.305	−0.18	0.543	0.01	0.586	0.01	0.070	0.01	0.470	0.01	0.099
Flavan-3-ol (mg)	0.05	0.862	−0.06	0.799	0.01	0.651	0.01	0.814	0.01	0.947	−0.01	0.436
Flavones (mg)	0.09	0.554	−0.40	0.211	−0.02	0.881	−0.33	0.061	0.08	0.709	−0.69	0.043
Flavonols (mg)	0.01	0.206	0.01	0.562	0.01	0.440	−0.01	0.048	0.01	0.598	−0.01	0.065
Flavonones (mg)	0.01	0.509	0.05	0.199	0.10	0.900	0.02	0.076	−0.01	0.682	−0.06	0.047
Anthocyanidins (mg)	−0.10	0.777	−0.05	0.159	0.01	0.695	−0.01	0.123	0.01	0.934	−0.03	0.053
Isoflavones (mg)	0.01	0.884	−0.02	0.294	0.01	0.755	0.01	0.180	0.01	0.286	0.01	0.125

^a^Models were adjusted by age, sex, physical activity, medication use, and obesity comorbidities. Models were not adjusted for total energy intake and dietary supplements because they are the DII® components. AST, aspartate aminotransferase; ALT, alanine aminotransferase; GGT, gamma-glutamyl transferase; NAFLD-FLS, Nonalcoholic Fatty Liver Disease Liver Fat Score; FLI, Fatty Liver Index; FIB-4, Fibrosis Index based on four factors. DII®, Dietary Inflammatory Index; hs-CRP, high-sensitivity C-reactive protein; IL-6, interleukin-6; TNF-*α*, tumor necrosis factor alpha.

**Table 5:** Regression analyses of the hepatic status parameters after 6 months of dietary intervention as dependent variables and changes in anthropometric, biochemical, and dietary factors as independent variables in the CD group^a^.

Variable changes (Δ)	Control diet group											
	NAFLD-FLS				FLI				FIB-4			
	Unadjusted		Adjusted		Unadjusted		Adjusted		Unadjusted		Adjusted	
	Β	*p* value	*β*	*p* value	*β*	*p* value	*β*	*p* value	*β*	*p* value	*β*	*p* value
Bodyweight (kg)	−0.05	0.819	−0.04	0.752	−0.03	0.605	0.03	0.599	0.10	0.750	−0.10	0.706
BMI (kg/m^2^)	−0.02	0.969	0.01	0.967	−0.01	0.956	−0.11	0.313	0.67	0.324	0.36	0.481
Fat tissue (%)	−0.19	0.503	−0.15	0.126	−0.05	0.423	−0.09	0.037	−0.07	0.845	−0.53	0.021
Visceral adipose tissue (l)	−0.14	0.748	−0.10	0.620	−0.01	0.900	0.05	0.525	1.36	0.094	0.66	0.123
hs-CRP (mg/l)	−0.17	0.350	−0.16	0.081	−0.01	0.849	−0.07	0.058	0.07	0.777	0.07	0.639
IL-6 (pg/mL)	−0.60	0.614	−0.78	0.247	−0.25	0.504	−0.61	0.020	−2.36	0.313	−0.08	0.928
TNF-*α* (pg/mL)	−1.16	0.609	−0.94	0.425	−0.31	0.562	−0.69	0.172	−5.78	0.136	−2.97	0.213
DII®	0.01	0.967	0.01	0.991	0.06	0.484	0.10	0.360	0.02	0.965	0.16	0.749
Energy (MJ)	0.01	0.694	0.01	0.591	0.01	0.566	0.01	0.188	0.01	0.638	−0.03	0.921
Proteins (%MJ)	0.01	0.997	−0.02	0.891	0.05	0.414	0.12	0.043	−0.30	0.381	−0.13	0.565
Total fat (%MJ)	−0.01	0.944	−0.01	0.891	0.01	0.631	0.03	0.331	−0.21	0.186	−0.19	0.179
Omega-3 (%MJ)	7.98	0.431	7.27	0.142	0.77	0.734	4.03	0.059	−13.45	0.354	−6.38	0.460
Total polyphenols (mg)	0.02	0.298	0.02	0.167	0.01	0.816	0.02	0.975	−0.02	0.584	−0.01	0.660
Flavan-3-ol (mg)	0.02	0.708	0.02	0.613	0.01	0.480	0.02	0.181	−0.11	0.205	−0.05	0.402
Flavones (mg)	−0.40	0.147	−0.42	0.027	0.03	0.547	0.03	0.584	−0.84	0.064	−0.58	0.074
Flavonols (mg)	−0.03	0.297	−0.03	0.118	0.02	0.535	−0.01	0.346	0.03	0.366	0.00	0.862
Flavonones (mg)	0.01	0.370	0.00	0.234	0.01	0.410	0.00	0.413	0.00	0.711	0.00	0.727
Anthocyanidins (mg)	−0.07	0.148	−0.07	0.012	0.01	0.605	−0.02	0.059	0.03	0.585	−0.01	0.848
Isoflavones (mg)	0.05	0.930	0.01	0.891	0.21	0.204	0.18	0.188	−0.98	0.288	−0.05	0.402

^a^Models were adjusted by age, sex, physical activity, medication use, and obesity comorbidities. Models were not adjusted for total energy intake and dietary supplements because they are the DII® components. AST, aspartate aminotransferase; ALT, alanine aminotransferase; GGT, gamma-glutamyl transferase; NAFLD-FLS, Nonalcoholic Fatty Liver Disease Liver Fat Score; FLI, Fatty Liver Index; FIB-4, Fibrosis Index based on four factors. DII®, Dietary Inflammatory Index; hs-CRP, high sensitivity C-reactive protein; IL-6, interleukin-6; TNF-*α*, tumor necrosis factor-alpha.

## Data Availability

The data used to support the findings of this study are included within the article.
